# Management Solutions for the Restructuring of Laboratories Associated to the Sentinel Services for Syphilis and Other STIs

**DOI:** 10.3389/fpubh.2022.841919

**Published:** 2022-04-29

**Authors:** Karilany Dantas Coutinho, Ricardo A. de M. Valentim, Geir Veras Vieira, Maíra Sidrim, Pedro Henrique Germano Evangelista, Laís Pereira de Oliveira

**Affiliations:** Laboratory of Technological Innovation in Health (LAIS), Federal University of Rio Grande do Norte (UFRN), Natal, Rio Grande do Norte, Brazil

**Keywords:** project management, quality, logistics, syphilis, health vigilance, SOP (standard operating procedure)

## Abstract

This article aims to develop management solutions to accompany the processes of acquiring and distributing equipment and/or materials needed for the restructuring of associated laboratories to the sentinel services (Sentinel Laboratories) for syphilis and other sexually transmitted infection in the scope of the “Syphilis No!” Project. To this end, we have taken steps to create an overview of the restructuring project for the sentinel laboratories, define the stages of project execution, monitor the implementation of the project, and elaborate a standard operating procedure for the delivery of equipment and/or materials to the sentinel laboratories. Among the results, we highlight: the detailed workflow for the process of public procurement through direct purchases or bidding; the workflow for storage of the equipment and materials; the standard operating procedures (SOP) for contact with the laboratories; and the SOP for delivery of items acquired for the sentinel laboratories.

## Introduction

Sexually transmitted infections (STI) are among the most common and transmissible infections affecting the health and lives of people around the world (1). The World Health Organisation (WHO) has estimated that, in 2016, a total of 376 million new curable STI took place within the population that is 15 to 49 years old. Of such cases, 6.3 million were caused by syphilis (2, 3). The registered syphilis numbers amounted to a rate of 1.5 cases for every 1,000 people around the world and 2 cases per 1,000 people in the Americas (3).

According to the Center for Disease Control and Prevention (CDC), in 2019, a total of 129,813 syphilis cases were notified in the United States, including 38,992 cases of primary and secondary (P&S) syphilis. After the period between 2000 and 2001 in which a historic low was observed, the rate of P&S syphilis has risen every year, with a focus on the period between 2018 and 2019 in which an increase of 11.2% was observed. Furthermore, an increase in the rate of syphilis was observed among men and women in all areas of the United States and among all ethnic groups (4). In Canada, one of the reasons that explain the recent rise in syphilis cases is the unequal accessibility within the healthcare system, including the lack of facilities in which to carry out testing for infections such as syphilis (5). In this sense, it is often hard to reach vulnerable populations due to factors such as a lack of housing, distrust in the healthcare system and the very lack of facilities for testing (5).

According to Zorrilla et al. (6), eliminating infectious diseases is a hard task because it depends on the intersection of science, coordinated actions and political intervention. Relating to the vertical transmission of HIV and syphilis, it is necessary to invest in research, training, extension and women-focused care. In May, 2016, the 69th World Health Assembly adopted a strategy for the global health sector regarding STI. This strategy includes the expansion of interventions and services based on evidence to control STI and diminish their impact as a health problem until 2030 - linked to the Sustainable Development Goals (SDG). Among these strategic points is the lowering of the global incidence of syphilis in 90% from 2018 to 2030 (7).

Between the years of 2010 and 2018, according to data from the 2020 Syphilis Epidemiological Bulletin (8–12), Brazil has registered a substantive amount of registered cases in all regions of the country. In September of 2017, after an extensive audit, the Brazilian Federal Court of Auditors (TCU) issued an operational report (*Acórdão* n° 2019/2017-PL) on the way the Brazilian federal government acted to control syphilis within the country. The findings of this report point to insufficient action on behalf of the Brazilian Ministry of Health (MoH). The TCU indicated that the MoH is the coordinator of the policies of investigation on the inequalities of performance of the services needed to control syphilis throughout the Brazilian municipalities. The findings also pointed to low efficiency in the measures adopted by the MoH to prevent primary sexual transmission in the population (11).

With this problem in mind and with the objective of developing a national policy of syphilis response in Brazil, the MoH declared, in 2016, a syphilis epidemic in the country. In this context, Brazil has developed, in 2017, a national and interfederative project to combat and respond to the syphilis epidemic. In the year 2018, the “Syphilis No!” Project was initiated throughout the national territory as a tool for health public policy induction to respond to the syphilis epidemic (8–11). The project was implemented through the Decentralized Execution Terms (TED 54/2017 and TED 111/2017) struck between the MoH and the Federal University of Rio Grande do Norte (UFRN). The “Syphilis No!” Project was coordinated through a work plan by UFRN's Laboratory of Technological Innovation in Health (LAIS/UFRN), in cooperation with other Brazilian universities, the MoH and the Pan American Health Organisation (PAHO) (9–11). The activities were distributed in 4 main axes: vigilance, management and governance, integral care and educommunication (13, 14). Within TED 111/2017, the main object focuses on applied research, structuring and improvement of the situation rooms for health vigilance in Brazil, and presents some work goals, among of which are: specifying and structuring technological environments to support the Secretary of Health Vigilance in the integration of health vigilance activities and syphilis response.

The MoH has defined the following objectives to prevent and control syphilis: developing actions to reduce morbimortality, define and indicate control measures related to transmission and to stop the transmission chain (9, 14). To effectively control syphilis, it is necessary to interrupt the transmission chain, which involves the detection and early and adequate treatment of the patient and their partners. Also, to prevent new cases, it is necessary to educate the populace, especially vulnerable groups, about the disease and ways to avoid it (13).

Faced with the necessity of improvement of health vigilance activities, the concept of health situation rooms (or intelligence rooms) was created, consisting in the consolidation of data regarding population health, making available information of epidemiological and operational indicators related to mortality, death vigilance, disease control and health promotion. This information favors the development of analysis which subsidizes the promotion of public policies and the evaluation of interventions made (8, 15).

To make the syphilis diagnosis it is necessary to associate the user's history, the clinical data and the detection of antigens or antibodies through laboratory tests. It is worth noting that this diagnosis must occur in two steps: trial and confirmation (8, 12). In this context, the MoH has identified a need to improve and started an action to restructure 17 (seventeen) local laboratories throughout Brazil in association to the sentinel services that centralize STIs-related medical services. The objective was to broaden access to testing and make the return of results more agile for the patient with the focus to better structure the direction of treatment, the monitoring of infections as well as providing necessary conditions for laboratorial problem-solving related to STIs, such as adequate storage of samples, making microscopy exams, bacterial cultures and molecular biology techniques.

Such local laboratories, in association with the sentinel services for STIs, have an important contribution to the access to testing for patients referred through the attention networks (16). With the integration of laboratorial data and other indicators of health vigilance management, there is a strengthening and broadening of response, which makes the system more resilient. Thus, technological environments alongside laboratories associated with the sentinel services will be important instruments to consolidate a network of syphilis response in Brazil.

Considering the necessities of each existing laboratory, the MoH has surveyed the equipment and materials needed to restructure the laboratories that support the services of the sentinel vigilance. Such items range from simple materials (e.g.,: swivel chairs, air-conditioning units and computers) to high-complexity equipment (e.g.,: thermocyclers, ultra freezers, biosafety cabins). The logistical processes for acquisition and distribution of this material were attributed to the LAIS/UFRN.

The logistical process of planning and implementing the flow of materials always focuses on guaranteeing that these items are delivered in the correct moment and in the intended quality, which are fundamental and extremely important practices that reflect customer satisfaction. The logistical process involves the receiving, storage, control, and distribution of materials within organizations as well as those that involve external environments, which include storage, distribution, and the delivery process to ensure that the products reach the clients in the best way possible. We also highlight that, in order to find the best way to find and distribute a product it is imperative to understand its characteristics and specifications (17–19).

The importance of such logistical operations is especially evident due to the increase in complexity of execution, the new strategies (e.g.,: utilization of management software), and even the outsourcing of logistical processes, seeking competitive advantages such as the improvement of productivity (20). With the increase in market demand and the evolution of product types to be delivered, especially high-complexity products with specific requirements for storage and distribution, outsourcing of logistical processes has risen as a way to bolster organizational cost-effectiveness. Also Arif et al. (21), the use of logistical service providers may be a more strategic option than performing one's own logistics, that is why outsourcing as a practice is becoming more common.

Faced with this context, the present article aims to develop management solutions to accompany acquisition, distribution and delivery processes for equipment and materials needed to restructure laboratories associated with the sentinel services, selected by the MoH within the scope of the “Syphilis No!” Project.

## Materials and Methods

**Figure 1:**
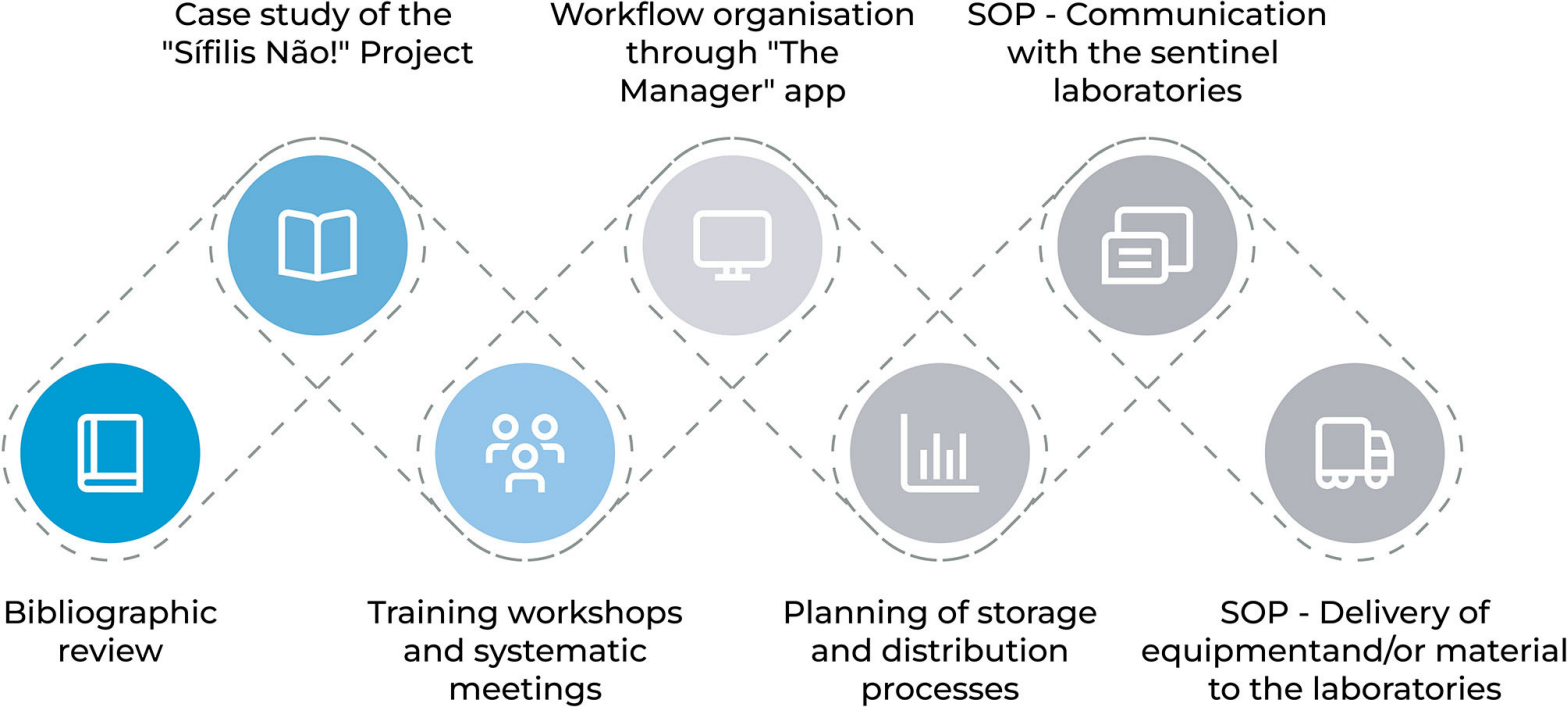
Steps for the methodological procedure.

Seven steps were executed in the development of management solutions to accompany the acquisition, distribution and delivery processes for equipment and materials needed to restructure the Sentinel Laboratories. Figure 1 presents the sequence of steps necessary to conduct the research.

The first step involved a bibliographic review. In this stage, the theoretical framework for the theme was explored so that it could be applied according to the necessities of the “Syphilis No!” Project and its execution phases. The second step concerns the study of the “Syphilis No!” Project and the Sentinel Laboratories to attempt to understand the priorities, demands, deadlines, among other activities included in the logistic process.

In the third step, meetings were conducted to discuss, define and plan the processes. The fourth step involved the organization of this entire process within a management and monitoring system called “The Manager” which was developed by the LAIS/UFRN team.

**Figure 2:**
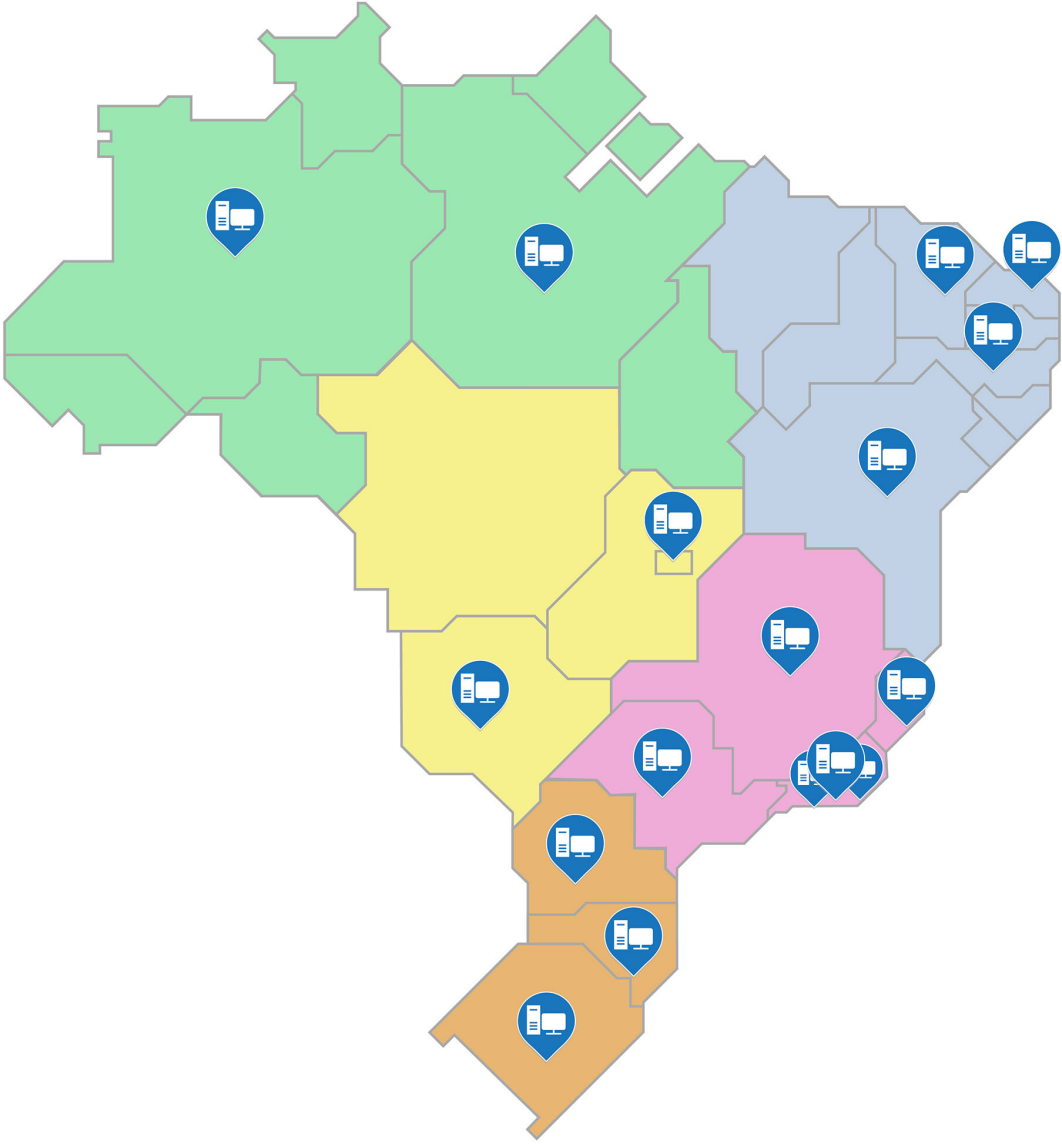
Distribution of sentinel laboratories contemplated in the scope of the “Syphilis No!” project.

For the fifth step, the processes of storage and distribution of acquired items were planned, including an analysis of the difficulties that may arise. At this stage, the MoH has carried out a survey of equipment and materials needed to restructure the laboratories and defined which of these laboratories would be contemplated considering the needs of each. Figure 2 shows the location of the 17 laboratories that were selected by the MoH to be restructured, these are located in 15 of the country's cities and encompasses the entire national territory.

Due to the amount of equipment and materials to be stored, distributed and delivered (a total of 339 equipment of big and small scale), a need to standardize some procedures through the application of SOP was identified.

After defining the distribution of equipment, the sixth and seventh steps involved the development of both SOP, one intended to confirm the information on the sentinel laboratories before delivery of equipment and materials; the other, intended to be applied at the moment of delivery of the items to the sentinel laboratories with the goal of standardizing and organizing the entire process, educate collaborators and producing documentation important to the project.

To define the steps and execution needs of the SOP, it was necessary to conduct systematic meetings with LAIS' logistics team, which is responsible for the process, then verify what steps should be contemplated.

According to Hollman et al. (22), it is recommended to make use of the Standard Operating Procedure (SOP) when work processes must be reproduced in the way that they were planned for multiple times or when the processes must adhere to conformity guidelines. To develop a SOP it is imperative to describe the activities and sequence of steps or procedure tasks through instructions following a gradual logic. The authors also affirm that the SOP must be written in a simple manner in order to be comprehensible to a multitude of people, including people from different fields of expertise.

## Results

### Steps of the Acquisition, Distribution and Delivery Process for Equipment and Materials

**Figure 3:**
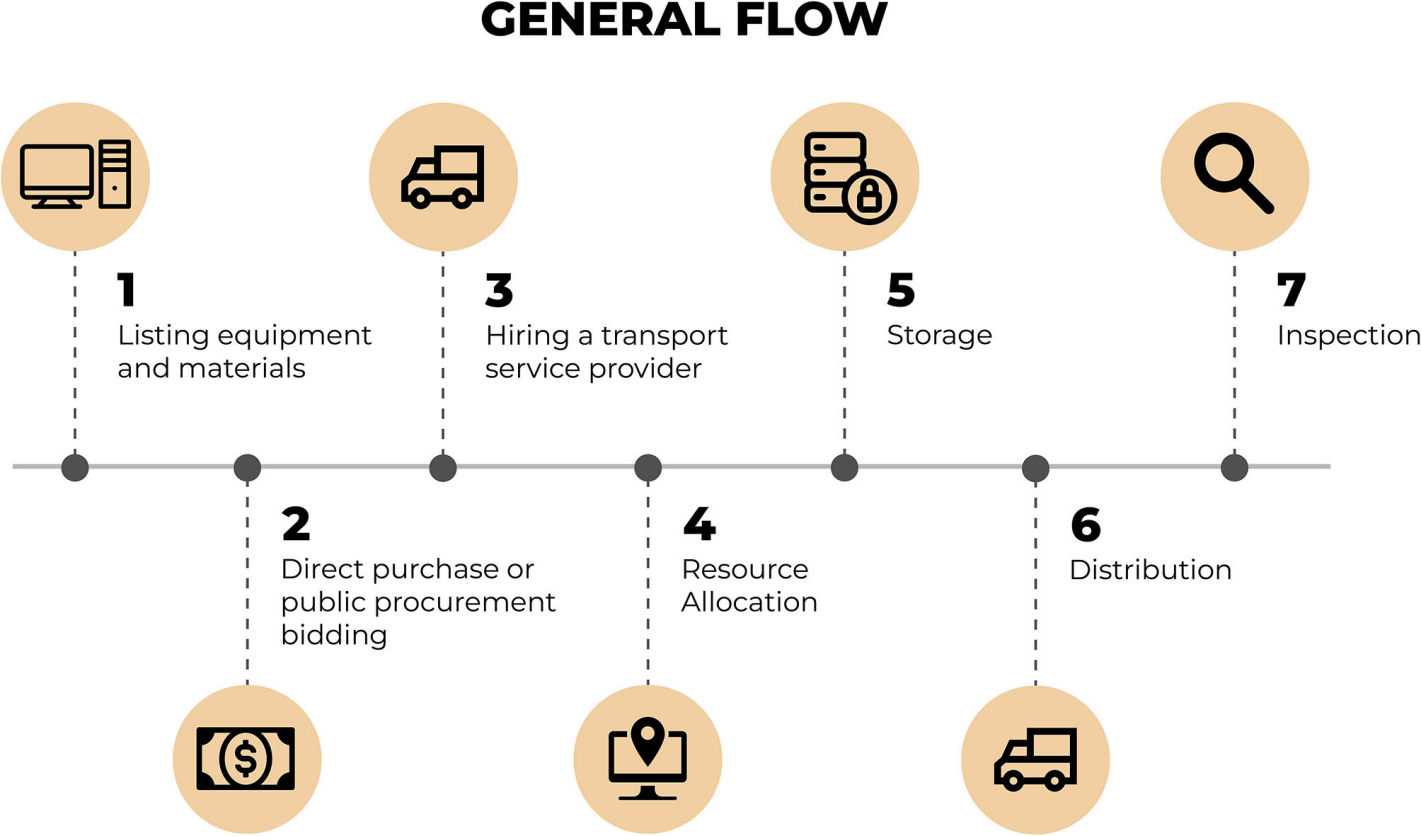
Flow of steps for the process of acquisition, distribution, and delivery of the equipment and/or materials. Source: graphic developed by the authors.

Figure 3 presents the sequence of steps followed from the definition of the list of equipment/materials up to the delivery of such items to the laboratories.

According to Figure 3, the first step of the flow was defined in the list with all equipment and materials to be acquired. The list was defined by the MoH and forwarded to UFRN containing the characteristics and quantities of equipment and materials that each laboratory should receive. In order to establish the list of equipment, the MoH has surveyed the 17 laboratories in the country and considered the individual needs of each for their restructuring. The items listed go from simple material (e.g.,: swivel chairs, air-conditioning units, computers) to high-complexity items (e.g.,: biosafety cabins, thermocyclers, ultra-freezers) as may be seen in the “Supplementary Materials” section.

The next stage involved the initiation of activities under the governance and coordination of the “Syphilis No!” Project that are related to the acquisition process (through direct purchase or bidding) until the distribution of all materials with the proper inspection.

The process was initiated with the acquisition of items listed and defined previously by the MoH. Such acquisition could be made through two scenarios: direct purchase or bidding, according to a decree that verses about the procurement of goods and the hire of services by support foundations.

Afterwards, the transport service provider was responsible for the distribution of all materials, being mindful of the specified requirements for the delivery of acquired items.

During the resource allocation phase, the payment was made for all equipment and materials acquired. From the moment of this acquisition, such items become a part of UFRN's estate due to the fact that the “Syphilis No!” Project is a project of the UFRN in cooperation with the MoH.

The following step involves the storage of equipment within UFRN's Directorate of Material and Assets. At this stage, items are duly stored in conditions that meet all of the necessary storage requirements so that they may go through the donation procedure of each of these items to their respective sentinel laboratories. Another necessary procedure that is made while items are in storage is the identification of assets, this involves the registration and identification of each item.

After the donation procedure, the distribution of equipment and materials to each sentinel laboratory occurs. For the distribution of equipment, the management of the “Syphilis No!” Project has opted, strategically, to outsource the transport service. This decision takes into consideration that Brazil is a country of continental proportions and that the distribution of 339 equipment and materials for sentinel laboratories involves countrywide coverage for 15 cities in 5 of the country's regions. Furthermore, the equipment and materials distributed have very high complexity and market value.

At the end of the distribution stage, the items distributed are inspected-which consists in the verification of whether these items were duly installed and if they are being utilized for the function previously established by the MoH and the “Syphilis No!” Project.

### Acquisition of Equipment and Materials: Direct Purchase and Bidding

In this section, the process of acquisition or purchase of equipment and material will be described. The purchase process was made through two processes: direct purchase or bidding (depending on the value of the equipment or material as specified in the decree that regulates the acquisition of goods and the hire of services by support foundations (23). In both cases, the Foundations of Teaching and Research, an entity administratively responsible for the “Syphilis No!” Project, is in charge of acquisitions according to the requirement of the project coordination and the MoH.

**Figure 4:**
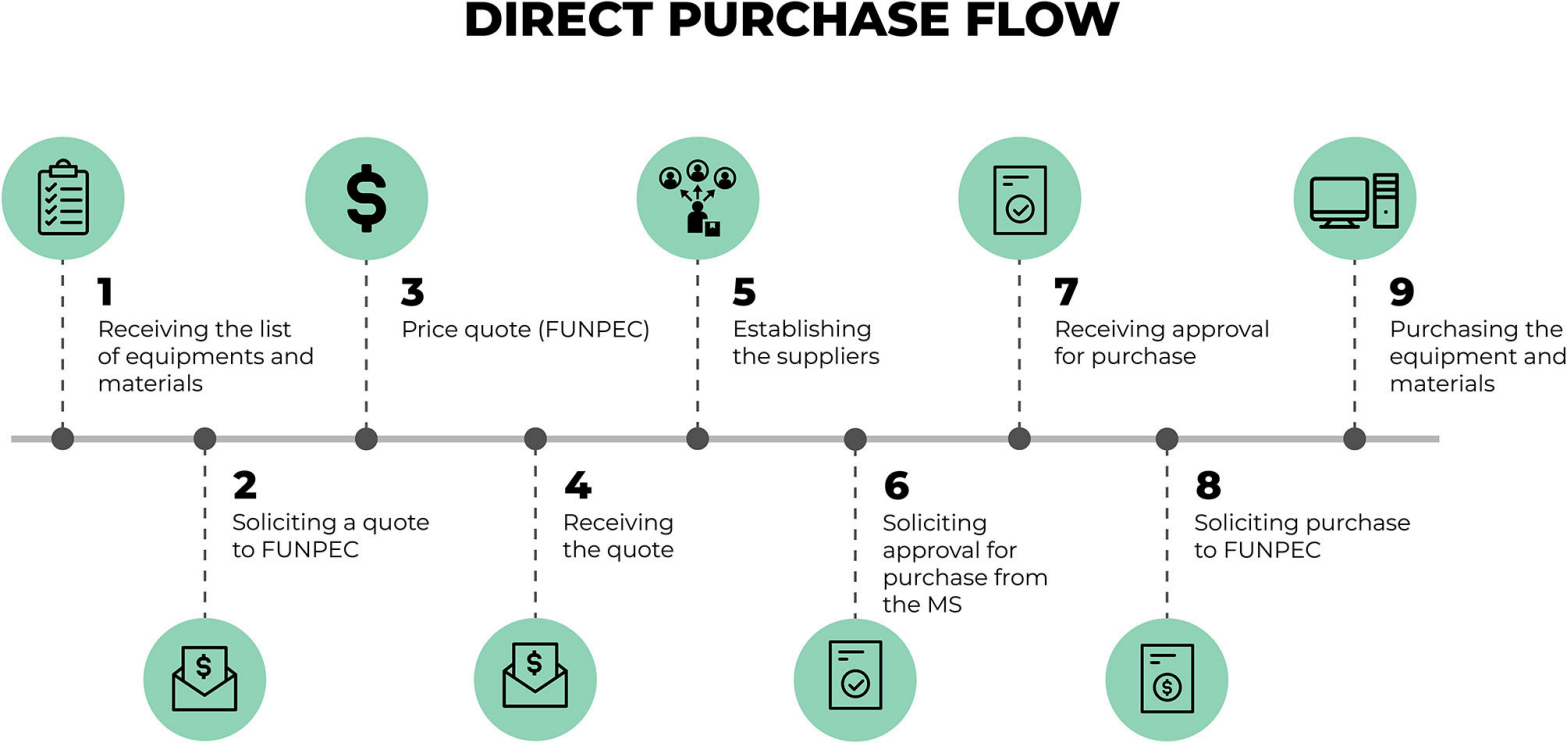
Detailed flow for procurement through direct purchase. Source: graphic developed by the authors.

Figure 4 presents the mapping of a workflow for acquisitions made through direct purchase without the need to go through bidding. To this end, the research foundation makes a market quote according to the specifications on the list of equipment previously defined by the MoH. After this survey, the process returns to the financial sector of the “Syphilis No!” Project. With this quote and the information on the materials that are collected, the financial sector of the project sends the information to the technical team of the MoH so that it may confirm if the quote and the equipment are in accordance with the requirements established by them. Should this information be confirmed, the solicitation is returned to the foundation so that it may proceed with the acquisition of the equipment and materials. This purchase is then carried out as well as the receiving and storage of items.

**Figure 5:**
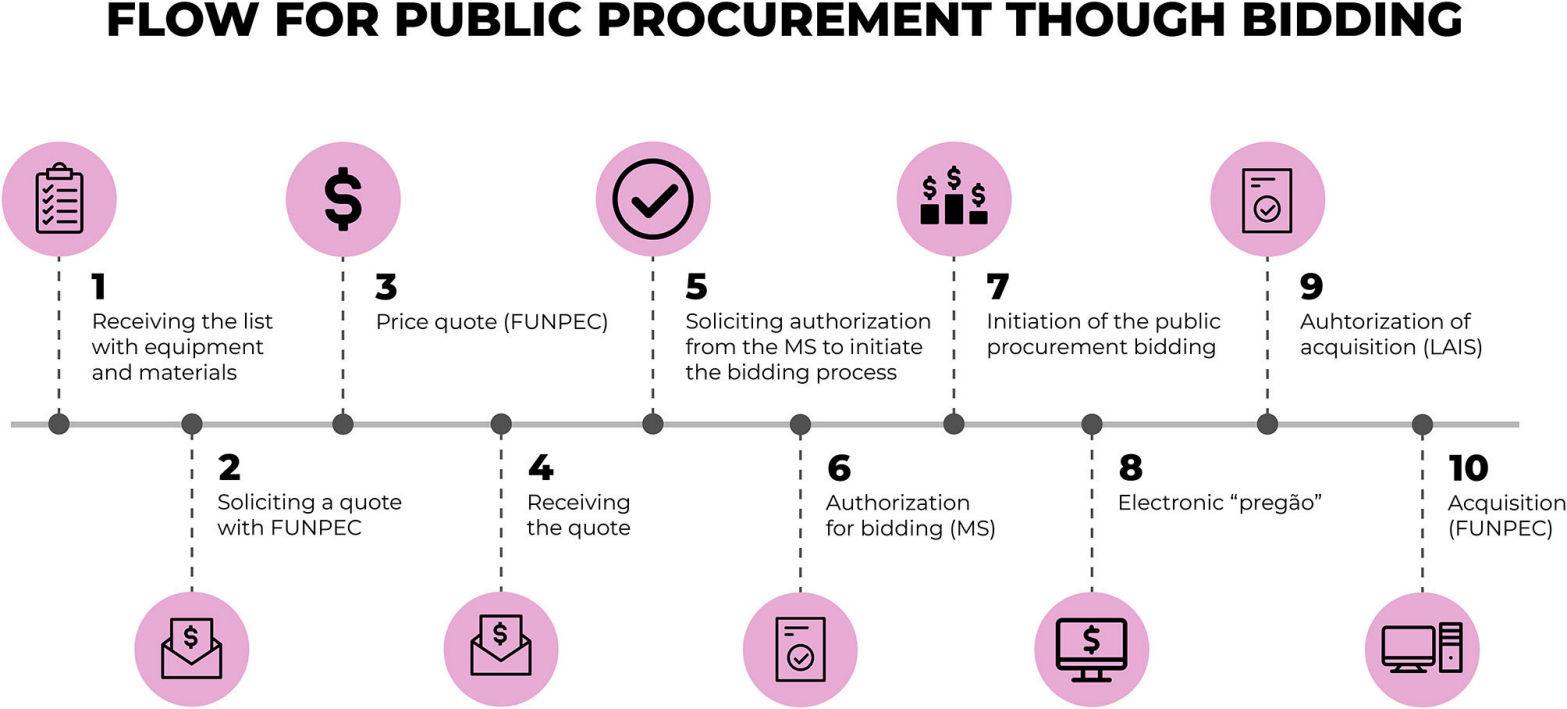
Detailed flow for procurement through bidding. Source: graphic developed by the authors.

In the situations in which the total value of the material to be purchased surpasses the limit established for direct purchases, according to the decree that regulates the acquisition of goods and the hire of services by foundations (23), it is necessary to procure such goods and services through a bidding process. Figure 5 presents the flow for the acquisition of equipment through the bidding process.

In the detailed flow for procurement through bidding, Figure 5, the financial department of the “Syphilis No!” Project requested a quote for the prices of products to the Foundations of Teaching and Research, which is administratively responsible for the Project. After making the quote, the Foundation forwards it to the Project's financial department in the same way that happens in the process for procurement through direct purchase. The financial department then forwards this information to the technical staff at the MoH in order for them to confirm if this quote and the equipment listed meets their requirements.

With all confirmations and authorisations duly registered both from the MoH and Project Coordination, the Foundation starts the bidding process by publishing a bidding notice that abides by the proper rules and regulations established in the decree (23) that verses about the acquisition of goods and the hire of services by foundations.

After going through the bidding process, the result is forwarded to Project Coordination and the MoH where the authorization is given to acquire the assets.

### Storage and Distribution of Acquired Items

The storage of acquired items, be it through direct purchase or bidding, is carried out in a safe manner within UFRN's Directorate of Material and Assets according to the necessary protocols for item inspection and technical specifications.

**Figure 6:**
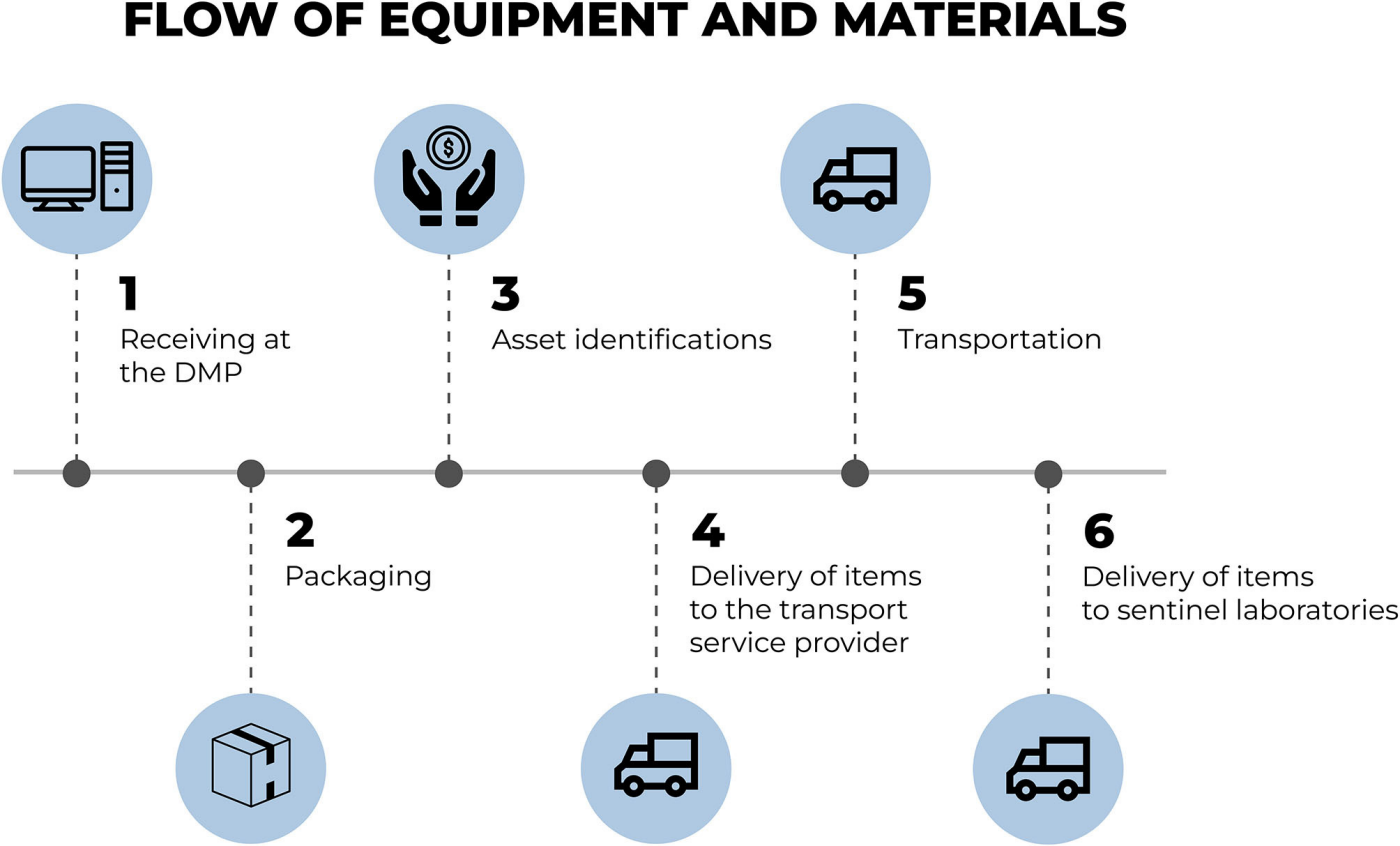
Flow for the storage of acquired equipment and materials. Source: graphic developed by the authors.

Figure 6 presents the flow for the storage of acquired equipment and materials. Upon being received for storage, the items are duly prepared, identified and registered (asset identification) as University assets. Furthermore, for reasons of monitoring and management, a tag is added containing information regarding the name and location of the sentinel laboratory to which the material is headed.

In the next stages, item distribution occurs through an outsourced transport service provider which delivers the equipment and materials to the locations of the sentinel laboratories.

To deliver this equipment to the laboratories, UFRN had to undergo an internal administrative process to donate these items in order for them to be removed from the UFRN's estate and become eligible to be received by the laboratories for which they are meant. This is due to the fact that the “Syphilis No!” Project is a project of the UFRN in technical cooperation with the MoH.

**Figure 7:**
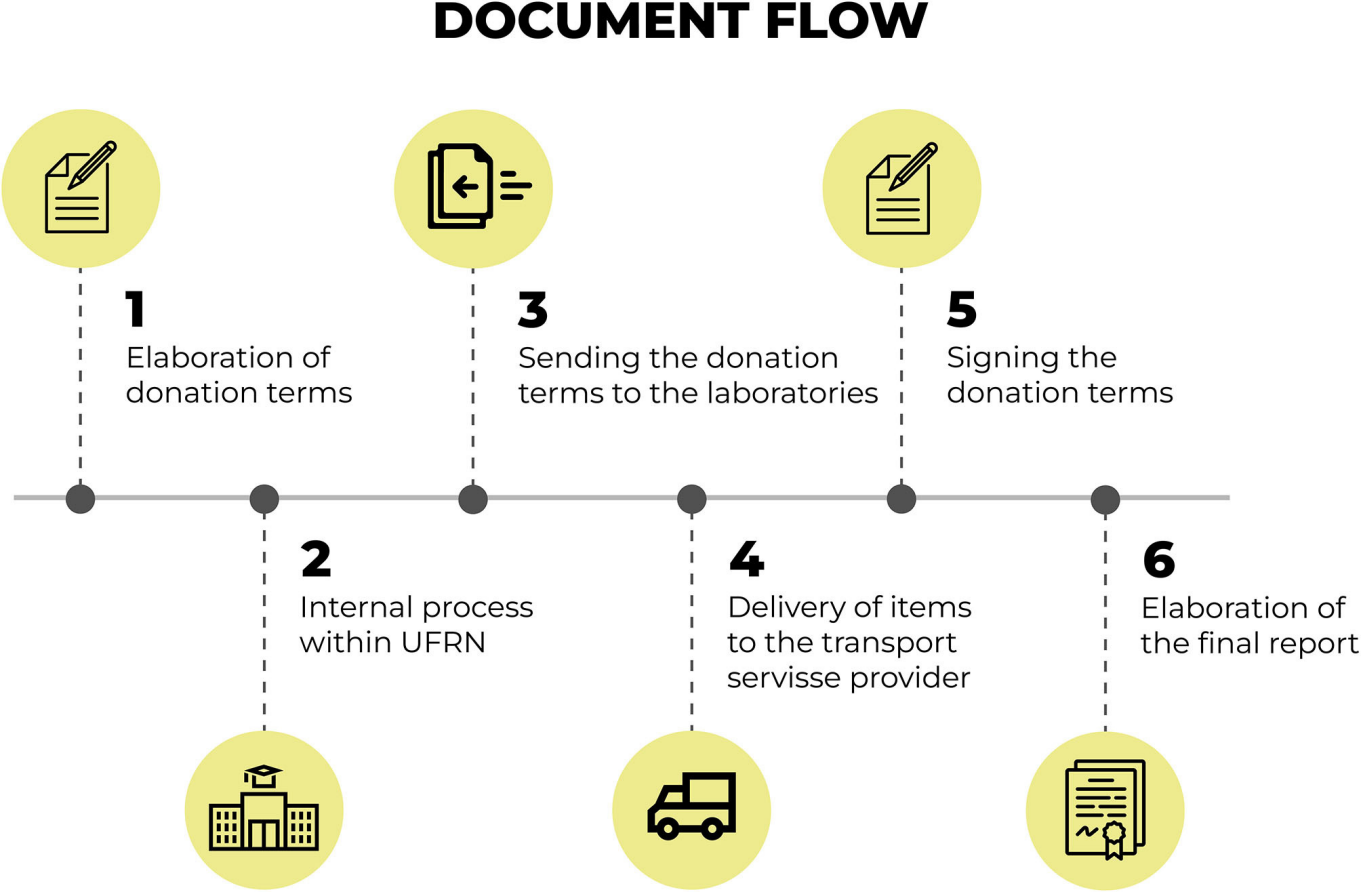
Flow for the documentation that permeates the distribution of equipment and materials. Source: graphic developed by the authors.

This fact has given rise to the need to execute other steps related to the documentation of the acquisition, distribution and delivery process for equipment and materials. Figure 7 presents the flow of documents that permeates the distribution of equipment and materials.

The flow for the documentation that permeates the distribution of equipment and materials, Figure 6, starts with the preparation of donation terms for the equipment and materials. Internal administrative procedures are carried out within the UFRN to propitiate the donation of equipment and materials for the sentinel laboratories.

Upon the end of the procedures, the donation terms are sent to the designated laboratories along with the terms of responsibility for the use of such equipment.

The terms are, then, signed by the people in charge of each sentinel laboratory upon delivery of the equipment and materials. All of these delivery procedures are accompanied by the team in charge of the “Syphilis No!” Project and a final report is made containing all necessary documentation.

### Inspection

Upon making the delivery of the equipment and materials to the people in charge of the sentinel laboratories, they are informed about the deadline of 30 (thirty) days to install the items delivered. It is, then, necessary to inspect the utilization of these items.

When all deliveries are concluded, the logistics team of the “Syphilis No!” Project makes random unannounced visits to the sentinel laboratories within the minimum timetable of 2 months after the delivery of items to their respective laboratories. The objective of this action is to verify whether items were properly installed and are being used for the functions previously defined by the MoH and the “Syphilis No!” Project.

For this stage it is necessary to make a technical visit to each laboratory for means of documentation, through photos, of the utilization of the materials and the identification tags to certify that the equipment present matches those delivered. The inspection report, summarizing all information, is, then, created.

After inspecting all sentinel laboratories, the Project's logistics team will elaborate a final report of all the acquisition, distribution and delivery process for the equipment/materials, thus, gathering all documentation produced during the process.

### Standard Operating Procedures

Envisioning the optimisation of processes, two SOP were produced: an SOP for communication with the laboratories and an SOP for equipment delivery. Both SOP are available in the “Supplementary Material” section.

The SOP for communication with the laboratories was developed with the aim to standardize all contact between the logistics team of the “Syphilis No!” Project and those responsible for the sentinel laboratories while also generating important documentation for the project. This contact occurs after the end of the internal procedures within UFRN, when the logistics team must contact the technical staff of the sentinel laboratories to explain how the delivery procedure will occur, to confirm information and to request the assembly of a team formed of laboratory staff to accompany the moment of delivery of the equipment.

The SOP for the equipment delivery strives to standardize and organize the delivery procedure of equipment and materials at the locations of the sentinel laboratories. All delivery procedures are accompanied by a team from the “Syphilis No!” Project.

The SOP were developed directly for the collaborators linked to the Project's logistic process with the aim of ensuring that all of these steps are carried out within the project of restructuring for the sentinel laboratories in an organized, systematic and standardized manner. Beyond contributing to the improvement of quality in a safe and efficient manner, the elaboration and implementation of the SOP also helps the process of organizational learning for the LAIS/UFRN staff.

The standardization of these stages is strategic for the “Syphilis No!” Project and LAIS/UFRN as it avoids the propagation of mistakes, reduces risk of wastage of time and financial resources while also helping the production of documentation necessary for the project, among which are audit documentation. Finally, the utilization of the SOP contributes to the continuous improvement of the process as well as stimulates organizational learning for the teams involved.

## Discussion

Within the vigilance axis of the “Syphilis No!” Project, the main goals are to strengthen syphilis testing throughout Brazil, improve the monitoring of infection indicators and facilitate the access of managers to this data. Local laboratories, in association with the sentinel services for syphilis and other STIs, play an important role in contributing with the access to patient testing for those who are suspicious of the disease, an aspect that quickens the return of the results to the patient and, thus, promotes a better direction for treatment and monitoring of infections (8). Integration of these results to the set of indicators of health vigilance is an important point for the coverage of the syphilis response network in Brazil because it strengthens the network which, in turn, contributes toward the improvement of health systems resilience in facing the syphilis epidemic.

Faced with this context, we highlight that the results described contributed to improve the efficiency of the acquisition and distribution processes for equipment and/or materials used in the restructuring of the sentinel laboratories. This result is one of the important factors for the control of the syphilis epidemic in Brazil, which will also stand as a legacy even after the conclusion of the “Syphilis No!” Project. Thus, within the actions carried out, we highlight the mapping of flows for the processes developed, from acquisition to delivery of the equipment and materials; and the creation of the SOP to contact the laboratories and to deliver the equipment and/or materials.

The mapping of flows is a powerful communication and planning tool while also serving as a basis from which collaborators may get to know in detail all steps of the process. In this sense, the elaboration of flows has contributed toward the comprehension of the process' stages by the team and served as an information multiplier for new collaborators and the project's documentation. The mapping of flows used may also be adapted for other public health urgencies that demand the structuring of sentinel laboratory networks.

With the development of the SOP, the logistics department of the “Syphilis No!” Project defined each step that must be carried out for the activities, thereby avoiding the propagation of mistakes and the wastage of resources. Furthermore, the implementation of the SOP aims to ensure that the necessary documents for future audits are timely produced with the correct quality. Thereby, we conclude that the utilization of this tool contributes toward the continuous improvement of the processes of LAIS/UFRN and, for this reason, may serve as a basis for future projects being clearly scalable and multipliable.

Finally, we conclude that the performance of this research contributes toward the strengthening of vigilance activities for syphilis and other STIs as this study has ensured more agility for the restructuring of the sentinel laboratories, a factor that has produced a positive impact for the syphilis response in Brazil.

## Data Availability Statement

The datasets presented in this study can be found in online repositories. The names of the repository/repositories and accession number(s) can be found in the article/supplementary material.

## Author Contributions

KC, GV, and MS concept and design the work and wrote the original draft. RV, PE, and LO contributed to the critical revision of the article. All authors have given their approval for this version to be published.

## Funding

This work was supported by the Syphilis No! Project from the Brazilian Ministry of Health (Grant TED No #111/2017).

## Conflict of Interest

The authors declare that the research was conducted in the absence of any commercial or financial relationships that could be construed as a potential conflict of interest.

## Publisher's Note

All claims expressed in this article are solely those of the authors and do not necessarily represent those of their affiliated organizations, or those of the publisher, the editors and the reviewers. Any product that may be evaluated in this article, or claim that may be made by its manufacturer, is not guaranteed or endorsed by the publisher.

## Abbreviations

CDC: Center for Disease Control and Prevention
MoH: Brazilian Ministry of Health
P&S: Cases of primary and secondary
PAHO: Pan American Health Organisation
SDG: Sustainable Development Goals
SOP: Standard Operating Procedures
STI: Sexually Transmitted Infections
TCU: Brazilian Federal Court of Auditors
UFRN: Federal University of Rio Grande do Norte
WHO: World Health Organisation
LAIS: Laboratory of Technological Innovation in Health
TED: Decentralized Execution Terms.
