# Supplementary material for: Management Solutions for the Restructuring of Laboratories Associated to the Sentinel Services for Syphilis and Other STIs
Source: Front Public Health. 2022 Apr 29;10:841919. doi: 10.3389/fpubh.2022.841919 (PMC9099240; doi:10.3389/fpubh.2022.841919)
Supplement: Supplementary file 1 [file Data_Sheet_1.PDF]

Standard Operating Procedure (Communication)	
Data from the person in charge of logistics	
Full name:	Time of contact:
E-mail:	Phone used:
Data from the sentinel laboratory	
Laboratory's name:	
Full name of the person in charge:	
E-mail:	Phone:

Instructions for the call

- Make a phone call to the person in charge of the sentinel laboratory (during commercial hours).
- Identify yourself while informing your name and your role in the project.
- Read the list of equipment and material that will be sent.
- Inform about the estimated date of delivery.
- Read the list of documents necessary for delivery.
- Request the formation of a receiving team composed of the person in charge of the laboratory and other staff that may have technical knowledge about the items sent.
- Explain the delivery procedure.
- Inform that an e-mail containing the same information will be sent for reasons of registration and documentation.
- Inform that the e-mail should be answered with a notice of receival in 3 business days.
- Request an e-mail address for further contact.

- Inform that the items may only be sent after this e-mail is answered.
- Upon the end of the call, take a screenshot of your phone with the call's information.

(Attachment) Screenshot with the call's information

Instructions for the e-mail

- Send an e-mail to the person in charge of the laboratory with the following text:

Dear Mr./Mrs. (Name of the person in charge of the laboratory).

My name is (full name).

As previously stated through our phone call, we are contacting you to inform about the estimated date of delivery for the equipment and/or material which (name of the laboratory) will receive through donation by the "Sífilis Não!" Project. The "Sífilis Não!" Project is a partnership between the Ministry of Health and the Federal University of Rio Grande do Norte (UFRN) through its Laboratory of Technological Innovation in Health (LAIS).

The estimated date of delivery for the equipment and/or material is: (date)

The person in charge of our team tasked with facilitating the delivery will be: (name and phone)

The list of equipment and/or material that will be sent as well as the donation terms are attached to this e-mail: (attach both files).

Please provide us with the name and the contact information for the person in charge of receiving the equipment and/or materials at the time of delivery.

We kindly ask you to notify us of the receival of this e-mail in the maximum timespan of 3 business days so that we may start the next stages. We also inform that the delivery may only take place after we obtain all information requested through this e-mail.

Kind regards,

(Full name, role in the project)

- Wait for the e-mail to be answered.
- If there is no answer after the deadline given, make another phone call to the person in charge of the laboratory informing about the need to answer the e-mail and give a new deadline for answer (2 business days) while warning that failure to answer halts the shipping of items.
- Verify if the answer to the e-mail contains: the laboratory's staff designated to receive the equipment and the confirmation that the person in charge of the laboratory is aware of the information given.
- Should any of this information be incomplete, answer the e-mail and inform about the necessity of completion of the pending information.
- Verify the new answer.
- Send an e-mail to the person in charge of the laboratory informing the date and the estimated time for delivery.
