# Supplementary material for: Management Solutions for the Restructuring of Laboratories Associated to the Sentinel Services for Syphilis and Other STIs
Source: Front Public Health. 2022 Apr 29;10:841919. doi: 10.3389/fpubh.2022.841919 (PMC9099240; doi:10.3389/fpubh.2022.841919)
Supplement: Supplementary file 2 [file Data_Sheet_2.PDF]

Standard Operating Procedure (Delivery)

Data from the person in charge of logistics

Full name:	Time of delivery:
E-mail:	Date of delivery:

Data from the sentinel laboratory

Name of the laboratory:	
Full name of the person in charge of the laboratory:	
E-mail:	Phone:
Full name of the commission member:	
E-mail:	Phone:
Full name of the commission member:	
E-mail:	Phone:

Instructions

- Identify yourself and your institution.
- Photograph the material while it is still in the transport service provider's vehicle.
- Confirm the presence of the person in charge of the laboratory and the other staff members designated to receive the material.
- Document through videos and photos the process of unloading the material.
- Organise all unloaded equipment and/or material and take photographs.
- Check, alongside the person in charge of the laboratory, the list of materials to be delivered.
- Deliver a copy of the list to the person in charge of the laboratory.

LAIS/UFRN
Av. Senador Salgado Filho - S/N - Natal/RN – Brasil
+55 84 3342-2250 - Ramal 140

- Document, through photos, the information and/or material and the preservation plaques.
- Inform, orally, about the 30 day deadline for equipment installation.
- Inform, orally, that the laboratory is now responsible for the equipment.
- Request a signature for the donation terms from the person in charge of the laboratory.
- Sign the donation term.
- Request the signature of two witnesses for the donation terms.
- Document, through photos and videos, the moment of signature of the donation terms.
- Fill out the receiving term.
- Document, through photos and videos, the filling out of the receiving term.
- Request a signature for the receiving term from the person in charge of the laboratory.
- Sign the receiving term.
- Request the signature of two witnesses for the receiving term.
- Request a signature for the receiving term from the delivery worker from the transport service provider
- Document, through photos and videos, the signature of the receiving term.
- Scan the donation and receiving terms, both properly signed.
- Prepare the delivery report, documenting all equipment and material delivered through photos, videos, emails, donation and receiving terms.
- Send delivery report to the project's coordination

LAIS
LABORATÓRIO DE INOVAÇÃO TECNOLÓGICA EM SAÚDE

UFRN
UNIVERSIDADE FEDERAL DO RIO GRANDE DO NORTE

LAIS/UFRN
Av. Senador Salgado Filho - S/N - Natal/RN – Brasil
+55 84 3342-2250 - Ramal 140

(Attachment) Images of the material inside the transport service provider’s vehicle
**(Attachment) Images of the material organised**
**(Attachment) Images of the preserved material**

(Attachment) Images of the donation term's signature

(Attachment) Images of the receiving term's signature
