# Supplementary material for: Management Solutions for the Restructuring of Laboratories Associated to the Sentinel Services for Syphilis and Other STIs
Source: Front Public Health. 2022 Apr 29;10:841919. doi: 10.3389/fpubh.2022.841919 (PMC9099240; doi:10.3389/fpubh.2022.841919)
Supplement: Supplementary file 3 [file Data_Sheet_3.PDF]

List of equipment and materials acquired.

Mechanical Agitator / Orbital Table
Vortex Mixer
Air-conditioning unit (17000 to 21000 BTUs - Split model)
Air-conditioning unit (9000 to 12000 BTUs - Split model)
Bain-Marie
Biosafety Cabinet Class II A2
Swivel Chair
Laboratory Centrifuge
Computer I5 or I7, 8GB RAM, SSD 240GB, Monitor 21.5'
Computer I5, 8GB RAM, HD 500GB, Monitor 21.5'
CO2 Greenhouse
Industrial Freezer (temp -20°C)
Quick-Freezing Freezer (temp -80°C)
Commercial Refrigerator with 2 Doors
Single Channel Micropipette (100-1000ul)
Single Channel Micropipette (1-10ul)
Single Channel Micropipette (20-200ul)
Multi Channel Micropipette with 12 Channels (20-200ul)
Multi Channel Micropipette with 8 Channels (20-200ul)
Laboratory Microscope (dark-field)

LAIS/UFRN
Av. Senador Salgado Filho - S/N - Natal/RN – Brasil
+55 84 3342-2250 - Ramal 140 | editais@lais.huol.ufrn.br

Optical Microscope
No-Break (2000 to 3000va)
Real-Time PCR
Electrophoresis System
Photo Documentation System
PCR Thermocycler
